# Welding and Joining of Titanium Aluminides

**DOI:** 10.3390/ma7074930

**Published:** 2014-06-25

**Authors:** Jian Cao, Junlei Qi, Xiaoguo Song, Jicai Feng

**Affiliations:** State Key Laboratory of Advanced Welding and Joining, Harbin Institute of Technology, Harbin 150001, China; E-Mails: jlqi@hit.edu.cn (J.Q.); songxg@hitwh.edu.cn (X.S.); feng_jicai@163.com (J.F.)

**Keywords:** welding, joining, titanium aluminides, microstructure, mechanical properties

## Abstract

Welding and joining of titanium aluminides is the key to making them more attractive in industrial fields. The purpose of this review is to provide a comprehensive overview of recent progress in welding and joining of titanium aluminides, as well as to introduce current research and application. The possible methods available for titanium aluminides involve brazing, diffusion bonding, fusion welding, friction welding and reactive joining. Of the numerous methods, solid-state diffusion bonding and vacuum brazing have been most heavily investigated for producing reliable joints. The current state of understanding and development of every welding and joining method for titanium aluminides is addressed respectively. The focus is on the fundamental understanding of microstructure characteristics and processing–microstructure–property relationships in the welding and joining of titanium aluminides to themselves and to other materials.

## 1. Introduction

Titanium aluminides have attracted great interest due to their high melting point, low density, high elastic ratio and good oxidation resistance [1,2,3]. Furthermore, intensive activity has been reported on the research and development of advanced titanium aluminides for high temperature structural applications during the last two decades [4,5,6]. Titanium aluminides are proposed as the most promising alternative light weight heat-resistant materials for heat-resistant steels and superalloys in aircraft turbine engines, airframes and automotive engines [2,5,7]. However, the poor room temperature ductility and fracture toughness has long inhibited their engineering application. Although there has been significant progress in the development of these alloys recently, particularly in improving the low ambient temperature ductility and workability [6,8,9], welding and joining of titanium aluminides is still one of the keys to their successful integration into high temperature aerospace and automobile applications [2,10,11,12,13].

Considerable attention has been paid to welding and joining of titanium aluminides, which consisted of γ-TiAl [14,15] and α_2_-Ti_3_Al [16,17] based alloys, to themselves and to other materials. Titanium aluminide joints have been fabricated by means of numerous approaches including fusion welding [18,19,20], brazing [21,22], diffusion bonding [23,24], friction welding [25,26] and reactive joining [27,28]. All these methods present their unique advantages and disadvantages in joining titanium aluminides, and are suitable for specific applications. This paper presents a brief review on current progress in the area of welding and joining of titanium aluminides. Particular emphasis has been put on the relationships between the microstructure and the mechanical properties. In addition, the highest joint strength and the corresponding joining parameters are summarized.

## 2. Brazing of Titanium Aluminides

Brazing, which is usually defined as a joining method with a molten filler metal and the solid substrates, can offer many advantages over other fusion welding methods such as a relatively low brazing temperature, the possibility of dissimilar materials and complex structures joining, high precision and better joint properties. In recent years, a number of investigations on brazing titanium aluminides have been reported which involve both conventional furnace brazing [29,30,31] and infrared brazing [14,15,17,32]. In general, titanium aluminides are suitable for brazing because most conventional filler metals can wet them well. However, it is necessary to optimize the brazing parameters to avoid the brittle interfacial products and the degradation of the substrates due to long holding times at high temperature.

The filler metal, brazing temperature, holding time and heating apparatus are the main factors that are used to optimize the brazing process. The selection of the filler metal is the fundamental step for brazing titanium aluminides. According to the previous literatures [14,33,34], it was noted that almost all kinds of filler metals were reported to successfully braze titanium aluminides. The main reason is the existence of active Ti element in the substrate, which usually served as an active element for brazing ceramics. The widely used filler metal consisted of Al-based [35], Ti-based [30,31,34,36], Ag-based [14,37,38,39] and Ni-based filler metal [33,40]. Considering the significant effect of filler metal on the microstructure, fundamental correlation between microstructure and brazing parameters varies with each filler metal. In this section, the present understanding of the microstructural evolution and corresponding joint properties using different kinds of filler metal is reviewed.

### 2.1. Brazing of Titanium Aluminides Using Ti-Based Filler Metal

Ti-based and Ag-based filler metals were the most commonly used for brazing of titanium aluminides [15,17,31,41]. Although Ag-based braze alloys exhibit good ductility and play the role of the buffer for the residual stresses, most alloys have low tensile strength and limited creep strength above 400 °C as compared with the Ti-based filler metal [32]. Commonly, the sound joints were fabricated with Ti-based filler metals because of the good chemical and physical compatibility between filler metal and titanium aluminides substrates.

The development of the Ti-based filler metal has attracted extensive research interest due to the potential engineering and scientific importance. Therefore, a series of Ti-based filler metal has been developed and utilized to braze titanium aluminides. The compositions of the Ti-based filler metal and the corresponding physical parameters are listed in Table 1. It is noted that most of filler metals contain Ni element, which is beneficial for improving the ductility of the brazing seam [22,29,30,42]. The addition of Cu element causes a significant decrease in the melting point and improves the flow ability of the Ti-based filler metal [17,32,34,43]. The Ti-Cu-Ni filler metal with the composition of 70%Ti-15%Ni-15%Cu (wt%) is the commonly used Ti-based filler metal and has successfully realized the brazing of TiAl. Nb or V elements were added into the filler metal to improve the compatibility with the Nb or V rich titanium aluminides, respectively [30,42]. The addition of B element can modify the mechanical properties of the brazing seam to form a gradient transition structure in the case of joining TiAl to C/SiC composite substrate [44].

**Table 1:** A summary of the composition of Ti-based filler metal for brazing titanium aluminides.

Filler metal	Composition (wt%)				Solidus temperature (°C)	Liquidus temperature (°C)	Brazing temperature (°C)	References
	Ti	Ni	Cu	Other				
Ti-Ni	67	33	–	–	942	980	980–1000	[33]
Ti-Cu-Ni	60	25	15	–	901	914	930–970	[17,32]
	70	15	15	–	910	960	1100–1200	[17,22,31,32,34,36,43]
Ti-Ni-Nb	31	38	–	31-Nb	–	1148	1160–1260	[42]
Ti-Ni-V	34	42	–	24-V	–	1142	1180–1260	[30]
Ti-Ni-B	33	64	–	3-B	–	1120	1180	[44]
Ti-Ni-Cu-Zr	52	23	12	13-Zr	809	858	900	[29]

**Figure 1:**
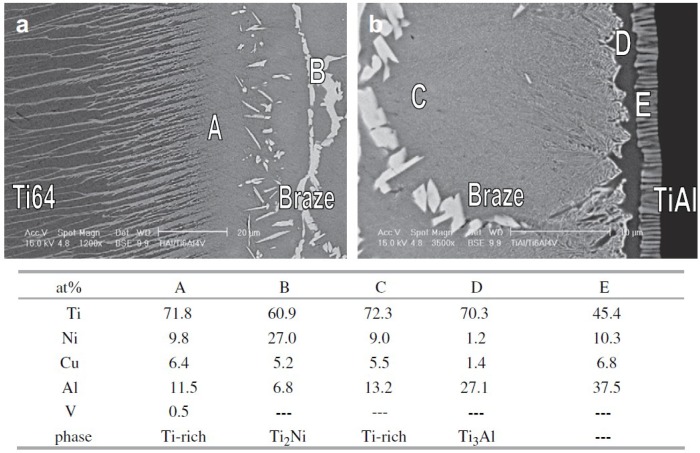
Scanning electron microscope (SEM) backscattered electron images (BEIs) and energy dispersive spectroscopy (EDS) chemical analysis results of infrared brazed Ti-6Al-4V and Ti50Al50 using Ti-15Cu-25Ni braze alloy at 970 °C for 300 s, (**a**) in the Ti-6Al-4V side; (**b**) in the TiAl side. (Reprinted with permission from [32]. Copyright 2008, Elsevier.)

As one of the rapid isothermal techniques, infrared vacuum brazing has been proved to be a useful way to join TiAl as a result of its short holding time at high temperature [32]. Although every filler metal mainly contains Ti element, various alloy elements of the substrate result in the different interfacial microstructure of the brazed joint. The microstructure characteristics attain great interest because the joint properties are determined by the microstructure. For Ti-15Cu-15Ni (wt%) filler metal, the interfacial reaction layer adjacent to the Ti50Al50 substrate is γ-TiAl/α-Ti/α + β/β-Ti/Residual liquid-phase (heated at 1150 °C for 30 s) and γ-TiAl/α-Ti/α_2_-Ti_3_Al/β-Ti/Residual liquid-phase (heated at 1150 °C for 60 s), respectively [43]. If the substrate was changed to Ti-48Al-2Nb-2Cr (wt%) alloy, the continuous α_2_-Ti_3_Al layer and wider columnar α + β two-phase zone tended to form due to the existence of Nb and Cr atoms, which served as the β-stabilizers of Ti-based alloys and lowered the proper temperature of α_2_-Ti_3_Al phase [34]. It is worth noting that both the original α-Ti phase and the γ-transformed α-phase existed, and both α-phase regions transformed into ordered α_2_-phase during cooling. When brazing of Ti50Al50 to Ti-6Al-4V using Ti-15Cu-15Ni (wt%) and Ti-15Cu-25Ni (wt%) filler metal at 970 °C for 300 s, the joints are mainly composed of Ti-rich, Ti_2_Ni and interfacial Ti_3_Al phases, as shown in Figure 1 [32]. When the holding time is prolonged to 600 s and 900 s, the phases in the brazed joint are similar to those of Figure 1. The amount of Ti_2_Ni phase is decreased with the increase of the holding time and the brazed joint for 900 s is free from Ti_2_Ni phase. However, when brazing Ti_3_Al substrate to Ti-6Al-4V using the same filler metal, the brazed joint only consists of primarily Ti-rich and Ti_2_Ni phases, and there is no interfacial reaction phase between the braze alloy and Ti_3_Al substrate [17].

The interfacial microstructure of furnace brazed TiAl joints is a little different from that of infrared brazed joints because of the change in reaction time. For example, for Ti-46Al-4(Cr, Nb, B) (wt%) furnace brazed joint, the reaction layer consisted of α_2_-Ti_3_Al phase and ternary τ_4_ phase in the middle of the joint [31]. For Ti-47Al-2Cr-2Nb (wt%) substrate, the joint can be divide into three distinct reaction layers: α_2_-Ti_3_Al + Ti(Ni,Cu)Al/α_2_-Ti_3_Al/α_2_-Ti_3_Al + Ti_2_(Ni,Cu) + Ti(Ni,Cu)_2_Al [22]. It should be noted that the Ni and Cu in the filler metal reacted with the TiAl substrate to form Al-rich compounds in the center of the joint. The results indicate that the long high-temperature holding in the furnace brazing process is beneficial for the solution of the Al in the substrate and reaction with Ti-Cu-Ni filler metal to form Al-rich compounds. The interfacial α_2_-Ti_3_Al layer was also reported by Dong *et al.* [29] when brazing Ti-48Al-2Cr-2Nb (wt%) alloys using Ti-Ni-Cu-Zr filler metal.

The addition of Cu into Ti-Ni based filler metal leads to the decrease of the melting point, but the high temperature application of the brazed joint is relatively limited at the same time. Therefore, the Cu-free Ti-Ni based filler metals were developed for brazing of TiAl. The Ti-47Al-8Nb (wt%) brazed joint using Ti-Ni filler metal mainly consists of Ti_2_Ni phase [33]. Furthermore, the TiNi-Nb and TiNi-V filler metals were designed to braze Nb-rich and V-rich TiAl substrate, respectively [30,42]. The joint mainly contained B_2_ phase (B_2_ phase with body-centered cubic structure is the ordered structure of the β phase due to the addition of alloying elements such as Nb or V) and the τ_3_ phase when brazing Ti-42.5Al-9V-0.3Y (wt%) alloys using TiNi-25V filler metal. When brazing Ti-45Al-5Nb-(W,B,Y) (wt%) alloys using Ti-Ni-Nb filler metal, the microstructure of the joint brazed at 1180 °C for 600 s was free of common structural imperfections such as interfacial microvoids, shrinkage cavities and microcracks. Combined with the EDS results, it was concluded that new intermetallics of O-Ti_2_AlNb and τ_3_-Al_3_(Ti,Nb)_2_Ni phases formed in the joint. When brazing Ti-46Al-0.5Si-0.5W (at%) alloys using TiH_2_-50Ni-2Si (wt%) powder braze alloys, the α_2_-Ti_3_Al/TiAlNi_2_/α_2_-Ti_3_Al + Ti_5_Si_3_ layered structure was observed in the joint [21]. There is no obvious microstructural difference between the TiAl joint using brazing foils and using brazing powders if the chemical composition is uniform. In practice, the application of brazing foils is limited for complex joint configurations in spite of the contaminants of binder residue for brazing powders. Thus, it is meaningful to manufacture Ti-based brazing powders owing to their convenience for application.

**Figure 2:**
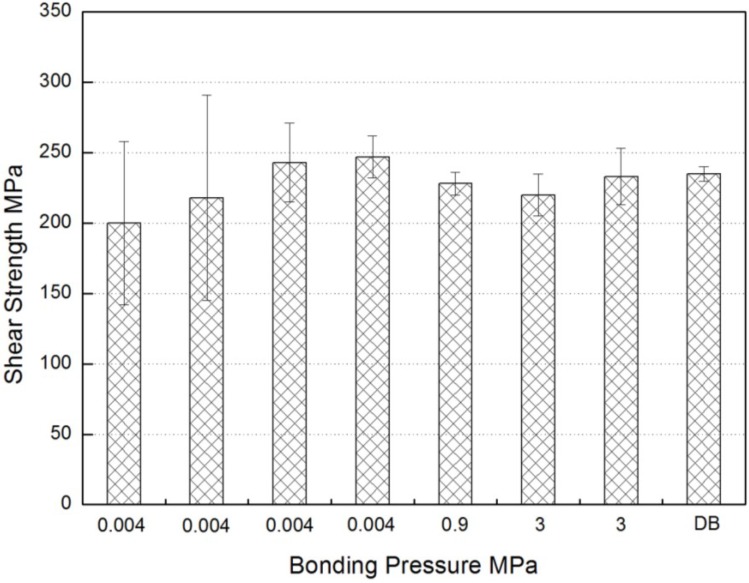
Effect of gap size on the shear strength of joints between γ-Met sheets, brazed with Ti-Cu-Ni powder at 1040 °C for 600 s. Data from a solid state diffusion bond (1050 °C for 14.4 ks with 40 MPa pressure) is also shown. The error bars represent two standard deviations from the average value. (Reprinted with permission from [31]. Copyright 2004, Elsevier.)

Although the applied pressure has a strong influence on the brazed joint, few investigations on the effect have been reported for brazing titanium aluminides. Most of the studies only point out that a light pressure such as 0.2 MPa [21] or 0.02 MPa [42] was applied to maintain sufficient contact at all bonded surfaces. Clearly, the liquid filler metal tends to flow out the joint and the thickness of the brazed seam becomes lower with the increase of pressure. However, minimal value existed for the thickness of the brazed seam because it is impossible to extrude all the filler metal. The effect of the applied pressure on the shear strength of the TiAl brazed joint using Ti-Cu-Ni filler metal is shown in Figure 2 [31]. It was noted that there was little difference in the strength values (220–230 MPa) or in the standard deviations (3.4–7.6 MPa) at the two higher pressures (0.9 and 3 MPa). When the bonding pressure of 0.004 MPa was applied, the strength decreased and the standard deviation significantly increased [31].

The mechanical properties of titanium aluminides brazed joint with Ti-based filler metal and the corresponding brazing parameters are listed in Table 2. Since the mechanical properties of the base metal to be joined differ from each other, it is meaningless to simply compare the shear strength of the brazed joint. For instance, when the TiAl alloys was brazed to C/SiC composites, the fracture occurred in the composites substrate and the joint was satisfied for the actual application even though the shear strength was only 105 MPa [44]. Therefore, if the fracture propagated in the substrate (as demonstrated in [34,42]), it suggested that the optimal microstructure and brazing parameters were attained. The interfacial reaction products in the brazed seam had a substantial influence on the joint properties and most of the fracture occurred near the brittle reaction phase, such as Ti_2_Ni phase [17] and τ_3_-Al_3_NiTi_2_ phase [42]. It was noted that most of the studies tested shear strength in spite the lack of exact test standards. In order to assess the interfacial joining quality, the key factor for shear test is to keep the area of the brazed seam smaller than that of the cross section of the substrate. Otherwise, the strength of fracture at the locations far away from the interface cannot reflect the joining quality accurately.

**Table 2:** The mechanical properties of titanium aluminides brazed joint using Ti-based filler metal.

Substrate	Filler Metal	Strength (MPa)	Brazing Parameters	Fracture Position	Brazing Method	References
Ti_3_Al/Ti-6Al-4V	Ti-15Cu-25Ni	304	930 °C/180 s	Ti_2_Ni	Infrared	[17]
	Ti-15Cu-15Ni	373	970 °C/600 s	Ti_2_Ni	Infrared	
Ti48Al-2Cr-2 Nb/40Cr steel	Ti-23Ni-12Cu-13Zr	32	900 °C/900 s	brazed seam	Furnace	[29]
Ti-42.5Al-9V-0.3Y	Ti-42Ni-24V	196	1220 °C/600 s	τ_3_-Al_3_NiTi_2_	Furnace	[30]
Ti-46Al-4(Cr, Nb, B)	Ti-15Cu-15Ni	220–230	1040 °C/600 s	–	Furnace	[31]
Ti50Al50/Ti-6Al-4V	Ti-15Cu-25Ni	189	970 °C/300 s	Ti_2_Ni, Ti_3_Al	Infrared	[32]
	Ti-15Cu-15Ni	280	970 °C/1200 s	–	Infrared	
Ti-48Al-2Nb-2Cr	Ti-15Cu-15Ni	319–322	1100–1200 °C/30–60 s	Substrate	Infrared	[34]
Ti-45Al-5Nb-(W, B, Y)	Ti-38Ni-31Nb	308	1220°C/600 s	Substrate	Furnace	[42]
Ti-43Al-9V-0.3Y/C/SiC composite	(TiH_2_-66Ni)97B3	105	1180 °C/600 s	C/SiC substrate	Furnace	[44]

### 2.2. Brazing of Titanium Aluminides Using Ag-Based Filler Metal

Compared with Ti-based filler metal, Ag-based braze alloys are featured with low brazing temperature and good ductility. It is reported that most Ag-based filler metals successfully braze titanium aluminides [13,14,15,37,38,41,45,46,47]. The joining of titanium aluminides is more difficult than many other engineering alloys because of the brittle substrate and reaction phases in the joint [15,39,46]. Consequently, the use of soft Ag-based filler metal is helpful for relaxing the residual stress generated in the cooling stage and obtaining successful joining.

Although numerous compositions of Ag-based filler metal were reported for brazing titanium aluminides, the common Ag-based filler metals can be divided into three series. Series I: Pure Ag filler metal [46]. The pure silver with the melting point of 961 °C was reported to easily braze TiAl intermetallics by Wu *et al.* [46]. Series II: Ag-Cu filler metal [15,45,47]. The most commonly used filler metal is Ag-28Cu eutectic filler metal due to the excellent wetting properties and the lowest melting point in Ag-Cu system. In other words, the addition of Cu in the Ag-based filler metal led to the decrease of the melting point, from 961 °C for pure Ag to 780 °C for Ag-Cu eutectic filler metal. Additionally, the flow ability of Ag-Cu eutectic filler metal is improved compared with pure Ag. Therefore, quite a few investigations on brazing titanium aluminides using Ag-Cu eutectic filler metal have been reported [15,47]. Series III: Ag-Cu-Ti filler metal [13,14,37,38,39,48,49,50]. It was noted that the brazing of titanium aluminides to other materials, such as steels [14,48,49] or ceramics [13,39], was successfully realized by the use of Ag-Cu-Ti filler metal. The addition of Ti element aims on the reaction wetting on the non-active materials. However, as a result of the Ti solution from the substrate, the direct brazing of titanium aluminides to ceramic is possible when the holding time is sufficient. In recent years, the development of composite filler metal, which was related to the addition of ceramic particles in Ag-Cu-Ti filler metal, was reported in brazing TiAl to Si_3_N_4_ ceramics [39]. The addition of the ceramic powders led to a decrease in the mismatch of the coefficient of thermal expansion and the Young’s modulus between the TiAl and ceramic substrates.

**Figure 3:**
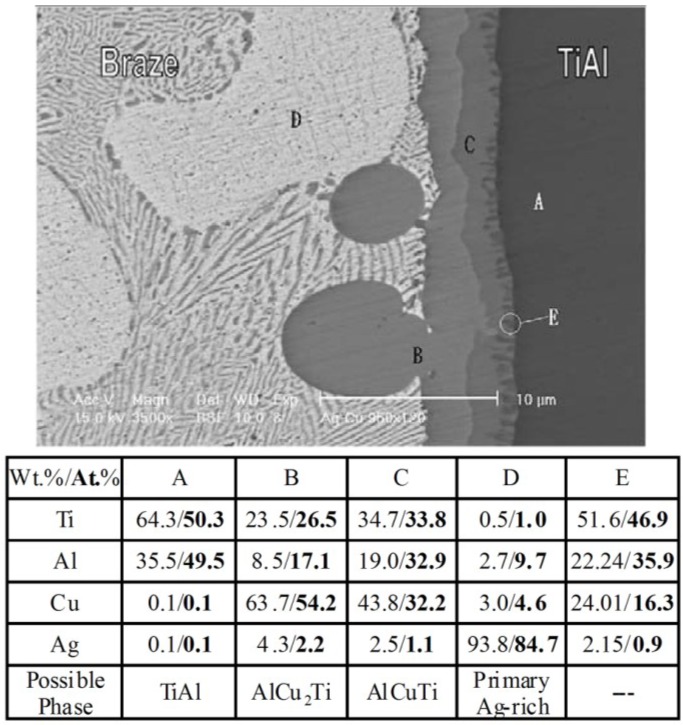
SEM BEI and EPMA chemical analysis results of the TiAl/BAg-8/TiAl specimens brazed at 950 °C for 120 s. (Reprinted with permission from [15]. Copyright 2003, Elsevier.)

The microstructure of the titanium aluminides brazed joint using Ag-based filler metal has been systematically analyzed. When pure Ag was used as filler metal, the reaction products of the TiAl brazed joint consisted of Ti(Al,Ag), Ti_3_(Al,Ag) and Ag-rich solid solution phases [46]. Since the Ag-rich phase dissolved much more Al than Ti, the consumption of Al led to the formation of Ti_3_(Al,Ag) phase. When the Ag-Cu or Ag-Cu-Ti filler metal was employed to braze titanium aluminides, the interfacial reaction phases adjacent to the substrate were mainly composed of AlCuTi and AlCu_2_Ti phases [14,15,47,48,49], as shown in Figure 3 (brazed at 950 °C for 120 s) [15].

When the holding time is very short (30 s), only AlCu_2_Ti layer was easily distinguished at the interface and large amounts of Ag-Cu eutectic structures were residual in the joint. When the holding time was increased to 45 s, the AlCuTi layer was observed at the interface and the primary Ag-rich phase was formed. With the increase of the holding time, the thickness of the interfacial AlCu_2_Ti and AlCuTi increased. The Ag-rich phase became predominant in the joint when the holding time was 180 s. Compared to Ti-Al-Ag compounds, the Ti-Al-Cu compounds are more stable and tend to be generated in the brazed system. Therefore, the Ti-Al-Cu compounds became predominant when the brazing temperature is higher and the holding time is long enough.

The mechanical properties of the titanium aluminides’ brazed joint using Ag-based filler metal are listed in Table 3. It was noted that the values of joint strength using pure Ag filler metal were similar with the TiAl substrate in the case of the brazing temperature of 1050 °C [46]. Taking the interfacial microstructure into consideration, the high joint strength derived from the ductile Ag-rich solid solution which buffered the residual stress. In addition, the Ti-Ag compound is not brittle, which was also beneficial for improving the joining strength [46]. The joining of titanium aluminides and structural steel with excellent shear strength is required in aerospace and automobile industries. Therefore, the brazing of titanium aluminides to steel are widely investigated using Ag-based filler metals by many researchers [14,37,41,48,49]. Although the strength of Ag-based filler metal was lower than that of Ti-based filler metal, the TiAl/steel brazed joint using Ag-based filler metal is higher. The formation of the carbide layer at the interface between filler metal and steel resulted in the impairment of the joint strength using Ti-based filler metal [14]. In other words, the use of Ag-based filler metal is beneficial for optimizing the interfacial reaction products.

**Table 3:** The mechanical properties of titanium aluminides brazed joint using Ag-based filler metal.

Substrate	Filler Metal	Strength (MPa)	Brazing Parameters	Fracture Position	Brazing Method	References
Ti-46Al-2Cr-2Nb/C/C composite	Ag-26.7Cu-4.6Ti	12.9	900 °C/600 s	TiC layer	Furnace	[13]
Ti-33.5Al-1.0Nb-0.5Cr-0.5Si/AISI4340	Ag-35.2Cu-1.8Ti	320	–	–	Infrared	[14]
Ti50Al50	BAg-8	343	950 °C/60 s	braze	Infrared	[15]
	Pure Ag	>385	1050 °C/30–180 s	substrate	Infrared	[46]
Ti-47Al/AISI 4140	AgCu + Ti	294	800°C/60 s	84% AlCu_2_Ti + AlCuTi layers	Infrared	[37]
TiAl48Cr2Nb2	Ag-28Cu	149	900 °C/60 s	–	Furnace	[45]
Ti-47.5Al-2.5V/Si_3_N_4_ ceramics	AgCu	124.6	850 °C/300 s	–	Furnace	[47]
Ti-48Al-2Cr-2Nb/35CrMo	Ag-Cu35.2-Ti1.8	320	870 °C/300 s	Al-Cu-Ti layer	Furnace	[48]
Ti-46.5Al-2.5V-1.0Cr/42CrMo	Ag-33Cu-4.5Ti	347	900 °C/300 s	AlCu_2_Ti phase	Furnace	[49]

In contrast to conventional brazing, transient liquid phase (TLP) bonding is eminently suitable to the joining of components intended for demanding high temperature service [51]. Although originally intended for Ni-based alloys, the reach of TLP bonding has now extented to a variety of materials including ceramics and intermetallics. Combining isothermal solidification with a subsequent solid state homogenisation treatment, TLP bonding offers the possiblity of producing bonds with similar microstructure and mechanical properties to the substrate, which is suitable for joining titanium aluminides. The first step for TLP bonding is to design a suitable interlayer. The reported interlayer included Ti/Cu foil [52], Ti/Ni foil [52,53], Ti/Fe foil [52], Cu + TiAl powder [54,55] and Ti-Cu-Ni foil [56]. Zou *et al.* [56] reported the transient liquid phase bonding of Ti-22Al-25Nb (at%) with Ti-15Cu-15Ni (wt%) and the optimal microstructure mainly consisted of matrix B_2_ phase, a few amounts of α_2_ phase and O phase. The tensile strength of the joint bonded at 970 °C for 5.4 ks was equal to 93% base metal. It was noted that in most of the TLP interlayer containing Ti and Cu elements it was easy to realize the isothermal solidicication and solid state homogenisation for titanium aluminides substrate. The holding time for solid state homogenisation is usually much longer than that for isothermal solidicication. Therefore, most studies on TLP bonding of TiAl was related to the post bond heat treatment with long holding time, for example heated at 850 °C for 18 ks [52].

**Figure 4:**
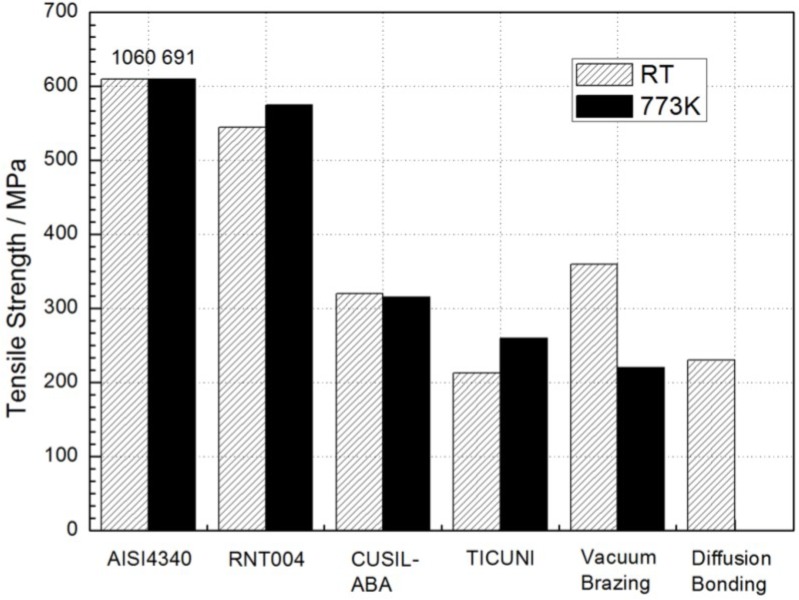
Tensile strength of TiAl joints. (Reprinted with permission from [14]. Copyright 1997, Elsevier.)

Considering most of the applications of the titanium aluminides’ joint performed at high temperature, the testing of the joint strength at high temperature becomes an increasingly important issue. However, only few studies [14,41,49] reported the strength at high temperature. The tensile strength of the TiAl/steel joint at room temperature and at 500 °C was reported in Figure 4 [14]. The strength of joint using CUSIL-ABA, 320 MPa at room temperature and 310 MPa at 500 °C, is considered sufficient for practical applications although the strength was lower than the TiAl and steel substrate. It was noted that the strength of the joint using Ti-Cu-Ni filler metal at 500 °C was slightly higher than that at room temperature [14]. This is possibly attributed to the relaxation of the residual stresses in the joint at the high temperature.

### 2.3. Brazing of Titanium Aluminides Using Other Kinds of Filler Metal

The Al-based filler metal was selected to braze titanium aluminides because of low density, low melting point and moderate corrosion resistance. Shiue *et al.* [35] pointed out that all infrared brazed joints using pure Al filler were unbonded upon a cross section of specimens because of the extensive presence of stable TiAl_3_ phase at the interface. When BAlSi-4 filler metal was used, the reliable brazed joint can be obtained and the highest shear strength of 86.2 MPa was reached [35]. While the transient interfacial AlSi_3_Ti_2_ phase is only observed in the short brazing cycle, the Al_12_Si_3_Ti_5_ phase was stable and dominated the interface between braze and TiAl substrate. When the TiAl samples were brazed at 900 °C for 45 s, the joint contained complex interfacial microstructure. The matrix of the brazed seam was mainly comprised of Al-rich solid solution with minor Si and Ti contents. Especially, both AlSi_3_Ti_2_ and Al_12_Si_3_Ti_5_ phases were observed at the interface. With the increase of either the brazing temperature or holding time, the amount of AlSi_3_Ti_2_ phase is greatly decreased at the interface. If the joint is held longer than 120 s, AlSi_3_Ti_2_ phase disappeared completely and only Al_12_Si_3_Ti_5_ phases were residual at the interface.

For specimen brazed less than 120 s at 900 °C, the fracture propagated at the interface between Al_12_Si_3_Ti_5_ phase and filler metal. With the extension of the holding time, the fracture path shifts from the interface to the continuous Al_12_Si_3_Ti_5_ layer due to the increase of its thickness. Upon further holding time, the fractured location changed from Al_12_Si_3_Ti_5_ layer into the braze alloy itself [35]. Although no obvious defects were found, the strength of the joint using BAlSi-4 filler metal was relatively lower than that using Ti-based and Ag-based filler metal. The formation of brittle Ti-Al-Si ternary compounds resulted in the low strength of TiAl brazed joint. However, the Ti-Al-Si compounds on the TiAl substrate played a positive effect on the oxidation resistance. Xiong *et al.* [57] developed a simple and inexpensive method to modify the surface of TiAl by liquid-phase siliconizing by Al-12.87 wt% Si and Al-10 wt% Si filler metal, which resulted in the main siliconizing products of Ti_7_Al_5_Si_12_ and Ti_5_Al_12_Si_3,_ respectively.

**Figure 5:**
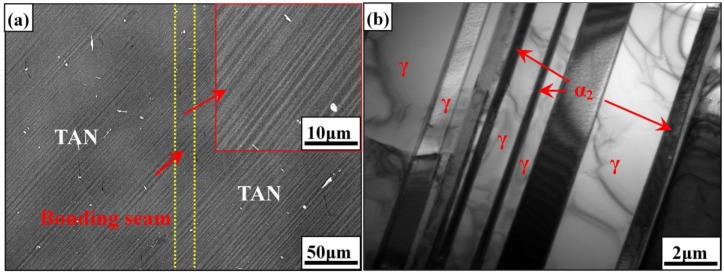
Interfacial microstructures of TiAl/Al/TiAl joint bonded at 1345 °C for 3.6 ks followed by stabilization at 900 °C for 1.8 ks and air cooling: (**a**) BEI and (**b**) transmission electron microscope (TEM) image (Reprinted with permission from [60]. Copyright 2014, Elsevier.)

Although the prior result [35] indicated that the brazing of TiAl intermetallics using pure Al filler metal is difficult, the application of pure Al filler metal is promising because no impurity element is introduced. Recently, the preparation of TiAl sheets was accomplished by accumulated roll bonding of alternating Ti and Al foils and corresponding heat treatment [58,59]. In fact, the method is a two-step process: (1) formation of TiAl_3_ phase and (2) gradual transformation from TiAl_3_ phase to γ-TiAl phase. Based on this, the reaction between the initial TiAl_3_ (the quantity was much smaller compared to the substrate when a thin Al foil was used) and TiAl substrate to form γ-TiAl phase is possible because there is a relatively large range for the content of Al in γ-TiAl phase. During the joining process, a heavy load and a post bond heat treatment (such as 1400 °C for 1.8 ks) are needed to ensure a sufficient contact and reaction at the interface. If the final interfacial microstructure is similar to that of base metal, high-quality bonding of TiAl alloys can be achieved. In other words, the titanium aluminides brazed joint with all Ti-Al intermetallics’ microstructure is promising for high temperature applications. Figure 5 shows the interfacial microstructure of TiAl with high Nb contents joint bonded at 1345 °C for 3.6 ks followed by stabilization at 900 °C for 1.8 ks and air cooling [60]. It was found that a sound joint with the fine full lamellar microstructure is obtained using pure Al filler metal. The bonding approach was simplified to a one-step process when the bonding assembly was directly bonded at the post bond heat treatment. The similar experiment was also carried out by inserting a pure Al sheet with a thickness of 15 μm as the filler metal [61]. The brazing specimens were heated at 900 °C for 3.84 ks and followed by heat treatment at 1300 °C for 3.84 ks. The TiAl_3_ phase formed in the brazing process and transformed to TiAl phase in the heat treatment process. The tensile strength of TiAl joints was the same as that of the based metal both at room temperature and at 600 °C.

The rapid development of brazing between steel and superalloys using Ni-based filler metal has motivated its application to titanium aluminides. However, a possible obstacle to the successful brazing of titanium aluminides using Ni-based filler metal is the formation of brittle Ti-Al and Ti-Al-Ni intermetallics. For the Ti-14Al-27Nb (at%) brazed joint using Ni-Cr8-Si5-B2-Fe2 (wt%) filler metal, the interfacial structures are believed to be TiAl_3_(TiB_2_)/TiAl_3_ + AlNi_2_Ti(TiB_2_)/Ni solid solution [40]. Tetsui *et al.* [33] also examined the microstructure of the TiAl brazed joint using Ni-15Cr-3.5B (wt%) filler metal with a brazing temperature ranging from 1005–1100 °C. While the AlNi_2_Ti phase and Ni solid solution are observed, the TiAl_3_ phase was not found at the interface. Clearly, the change of interfacial structures in the joint should be associated with the composition both in filler metal and in the substrate. The shear strength of the Ti_3_Al brazed joint using Ni-based filler metal was 240–250 MPa at 1323–1373 K/250–300 s, which was slightly lower than that using Ti-based and Ag-based filler metal [40].

Although both TiAl and Ti alloy contains Ti element, the wetting behavior of Ni based filler metal on the substrates differs from each other. Shapiro *et al.* [62] investigated the brazing titanium aluminides using Ni-27Ti-10Al and Ti-37.5Zr-15Cu-10Ni filler metal. The Ni-27Ti-10Al exhibited perfect wetting and gap filling of titanium-to-titanium joints. However, the TiAl based metal did not react with the Ni-27Ti-10Al filler metal at 1180–1200 °C. The negative effect can be explained by very high oxidation ability of hot-pressed TiAl manufactured from the pre-alloyed powder. Based on the interfacial reaction between TiAl and Ni-15Cr-3.5B filler metal [33], the formation of Ti-Al-Ni compounds stimulates the wetting behavior. Therefore, it is possible for the Ni-27Ti-10Al filler metal to wet the TiAl at the high enough brazing temperature, which was also demonstrated by the results in [62].

A novel approach to brazing of TiAl using Ni based filler metal is performed by using an electron beam as the heat source [63]. The electron beam has been defocused and has been moved fast and several times over the work pieces, which heats the braze material (Ni-7Cr-4.5Si-3.1B-3Fe wt%) evenly up to the brazing temperature of 1000 °C. The microstructural analyses were carried and six different zones of the base material via the transition zones up to different brazing zones were observed in the brazed joint. Despite no obvious improvement in the interfacial microstructure, the electron beam brazing of TiAl, which serves as a local heat method, was highly efficient and demonstrated a lower heat effect on the substrates.

Compared to the composition of conventional Ti-based and Ag-based filler metal for brazing titanium aluminides, it was noted that the Cu element was the only common content. Especially for Ag-Cu or Ag-Cu-Ti filler metal, the interfacial Ti-Cu or Ti-Al-Cu compounds became predominant when using high brazing temperature. Therefore, the application of Cu-based filler metal is possible for brazing titanium aluminides. Because the melting point of pure Cu is high, the P element is added to form Cu-P eutectic filler metal, which has been successfully used to braze Ti_3_Al intermetallics [64]. The optimum brazing temperature and holding time are 942–952 °C and 250–300 s, respectively. The interfacial structure of brazed Ti_3_Al joints with Cu-P filler metal is Ti_3_Al/Ti_3_Al(Cu)/TiCu/Cu_3_P/Cu solid solution. The maximum shear strength of the joint was 98.6 MPa when the brazing temperature was 950 °C. The fracture mainly propagated in the TiCu + Cu_3_P compounds and partially in the Cu solid solution [64]. It was noted that the joint strength was far less than that of the Ti_3_Al substrate, which derived from the thick and brittle reaction layers at the interface.

## 3. Diffusion Bonding of Titanium Aluminides

Sound joining of titanium aluminides to themselves and to other high temperature materials is one of the keys to their successful integration into high temperature aerospace applications [23]. To date, it is difficult for the fusion welding to avoid the solid-state cracks, and the strict preheating and post welding heat treatment is required. The application of the brazed joint in the high temperature is limited due to the low melting point of filler metal. Therefore, diffusion bonding has been considered to be more sutiable for joining of titanium aluminides [11,23,65,66,67,68,69].

### 3.1. Diffusion Bonding of Titanium Aluminides to Themselves

For the mechanism of the diffusion bonding, it is well acceptable that the bonding process can be considered as two stages: void formation stage and void shrinkage stage [70]. The surface roughness leads to the formation of void at the contact interface. When the pressure is applied, the contact area of interface will increase rapidly until it can support the pressure. Subsequently, the diffusion bonding with a high quality is achieved by plastic deformation and atomic diffusion in the void shrinkage stage with the extension of the holding time. Therefore, for the diffusion bonding of titanium aluminides to themselves, the main aim of the work is to obtain the similar microstructure and mechanical properties with the substrates. Since the residual thermal stresses were relatively small, the cracks were not main defects for joining the same materials.

**Figure 6:**
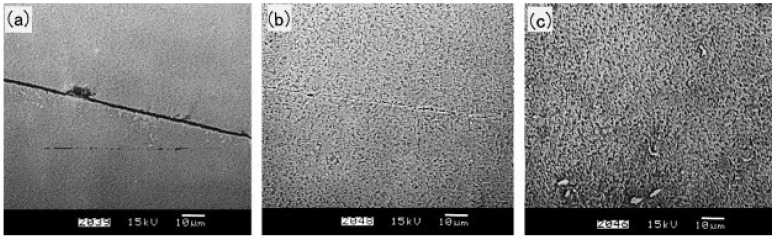
Microstructure of the interface at different stage uder a pressure of 60 MPa at 900 °C in 3.6 ks (**a**) the closure of long wavelength asperities; (**b**) the closure of short wavelength saperities and (**c**) the elimination of planar grain boundary interface and diffusion. Scale bars: 10 µm. (Reprinted with permission from [71]. Copyright 2001, Elsevier.)

The typical interfacial microstructue of the joint can be divided into three types. Type I: the unbonded regions are clear and the small amounts of voids remain at the interface, as shown in Figure 6a [71]. This type of microstructure is observed when the bonding temperature is low, the hold time is short or the bonding pressure is insufficient. The strength of joint with Type I microstructure is very low. Type II: no unbonded areas are observed but the bonding interface is clearly visible, as shown in Figure 6b [71]. With the increase of the bonding temperature or holding time, the unbonded areas disappeared by the deformation and atomic diffusion. However, due to the discontinuity of the grains in both sides, the bond interface is visible in the joint. Under some bonding parameters, the fine recrystallized gamma grains were formed at the interface resulted from asperity deformation on mating surface under the applied bonding pressure [23]. Type III: the original bonding line is invisible and the grains are continuous at the interface, as shown in Figure 6c [71]. The high bonding temperature leads to the sufficient interfacial diffusion and the formation of large grains at the bonding interface. The bonding line can only be distinguished through following the edges of the specimens. The joint with the Type III microstructure presents similar mechanical properties with the substrates. However, too high bonding temperature is not recommended because it may lead to the degradation of the titanium aluminide substrates.

**Figure 7:**
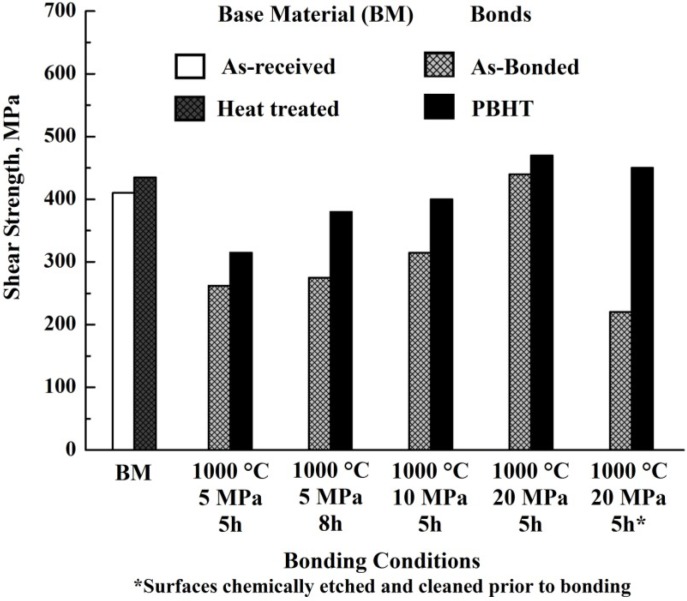
Results of compression overlap shear tests on γ-TiAl bonds in the as-bonded condition and after a post bond heat treatment (PBHT) at 1430 °C. Each strength value is average of at least four specimens. (Reprinted with permission from [11]. Copyright 1999, Elsevier.)

For conventional diffusion bonded TiAl joint, the loss in mechanical properties as compared to the base metal is still significant. Therefore, the effect of post bond heat treatment, which is the key factor to obtain the similar microstructure, has been studied in recent works [11,23,72]. Çam *et al.* [23] successfully realized the diffusion bonding of cast γ-TiAl alloys with and without post bond heat treatment (PBHT). The diffusion bonded joint under nonoptimized bonding parameters (*i.e.*, low bonding temperature, low bonding pressure or insufficient holding time) failed prodominantly along the bond interface and exhibited lower shear strength values. On the other hand, the bonds made at 1100 °C/20 MPa/10.8 ks mainly failed in the base metal away from the bond interface and the highest shear strength value (373 MPa) was obtained. It was noted that the bond line was almost invisible under optimum parameters. The increase of the strength can be explained by the fact that the cracks propagating through the big recrystallized grains became harder in contrast to the bonds with very fine γ grains at the interface. In addition, the PBHT improved the mechanical properties of all the diffusion bonded joints and all the bonds failed in the base metal after PBHT (Figure 7 [11]), which indicated the shear strength values of the joint were equal to that of base metal.

The strength of the titanium aluminide diffusion bonded joint and the corresponding bonding parameters are listed in Table 4. It was noted that the optimum parameters was not uniform because the physical and chemical properties of the base metal are different. In general, the optimal bonding temperature ranges from 1000–1100 °C and the post bond heat treatment is preferred. The high temperature and long holding time tends to induce structural changes and residual stresses at the interface and this is also a problem for industrial applications [73]. In order to enhance the joint properties and derease the bonding temperature, some effective methods are developed such as laser surface melting and interlayer addition. It was proved that the joint treated by laser surface melting presented a better bonding quality than that without treatment, and the ratio of shear strength of bonds to that of base metal incresed from 52%–64% [72]. The effect of interlayer on the bonding temperature are more significant than surface melting. An effective approach is to insert a hydrogenated Ti6Al4V foil as an interlayer for diffusion bonding of TiAl alloys [74]. The optimized bonding temperature deceased from 1200–850 °C when the same bonding strength was obtained. The results showed that the escape of the hydrogen in hydrogenated Ti6Al4V interlayer during the bonding process enhanced the plastic deformation and the diffusion ability of the alloy elements, which was beneficial for improving the bonding quality.

The application of Ni/Al multilayer foils as interlayer was a more exciting approach for optimizing the diffusion bonding parameters [66,73,75,76,77]. It was well known that a highly exothermic reaction occurred when Ni/Al multilayers transform into NiAl by a combustion synthesis reaction. In addition, the Ni/Al multilayer can further improve the diffusion bonding process by acting as a local heat source and promotes the diffusivity at the interface. In order to ensure a sound contact at the interface, the Ni/Al multilayers can be deposited on the TiAl substrate by magnetron sputtering. When the Ni/Al multilayers with a period of 14 nm were used, the shear strength for the bonds produced at 900 °C/5 MPa/3.6 ks reached 314 MPa, which is comparable to the strength using traditional diffusion bonding [23,72,74].

**Table 4:** The mechanical properties of titanium aluminide diffusion bonded joint.

Substrate	Interlayer	Strength (MPa)		Bonding Parameters	Fracture Position	References
		Joint	Substrate			
Ti-47Al-4.5 (Cr, Mn, Nb, Si, B)	–	373	430	1100 °C/20 MPa/10.8 ks	Substrate	[23]
		453	475	1100 °C/20 MPa/10.8 ks with 1430 °C/1.8 ks PBHT	Substrate	
Ti-48Al-3.7 (Nb, Cr, C)	–	388	1398 *	1000 °C/10 MPa/18 ks	Substrate	[65]
		580	–	1000 °C/20 MPa/3.6 ks with 1430 °C for 1.8 ks PBHT	Substrate	
Ti-46.5Al-2Cr-1.5 Nb-1V	–	384	468	laser surface melting 900 °C/60 MPa/7.2 ks	Substrate	[72]
γ-TiAl	14 nm period Ni/Al multilayer	314	–	900 °C/5 MPa/3.6 ks	Reaction layer	[73]
TiAl	hydrogenated Ti6Al4V interlayer	290	–	850 °C/15 MPa/900 s	Bonding zone	[74]
Ti-45Al-5Nb	14 nm period Ni/Al multilayer with Ti and Ni foils	38	–	900 °C/5 MPa/1.8 ks	Interface	[76]
Ti-47Al-2Cr-0.2Si	–	555 *	602 *	1000 °C/40 MPa/18 ks	Outside of bonding zone	[78]

* tensile strength, the other values are shear strength.

**Figure 8:**
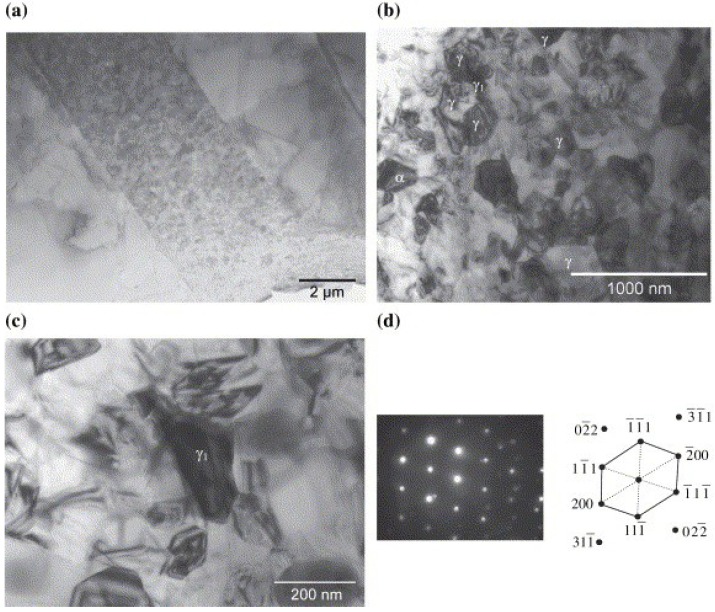
(**a**–**c**) TEM observations of nanocrystalline TiAl grains observed in the interface region of a joint processed at 1000 °C/50 MPa/3.6 ks; grains marked as γ are γ-TiAl and the one marked as α is α_2_-Ti_3_Al; (**d**) Selected area diffraction pattern of γ_1_ grain in (**b**,**c**). (Reprinted with permission from [82]. Copyright 2006, Elsevier.)

Recent experimental and computational works have provided significant insight about the interesting application of nano multilayers as interlayers in the joining process [24,73,79,80,81]. The use of nano multilayer as interlayers for diffusion bonding TiAl alloys was first reported by Duarte and Simões *et al.* [76,82,83,84]. A Ti/Al multilayer with a thickness of 2 μm and a period of 4 nm was deposited by dc-magnetron sputtering on the two surfaces to be joined. The effect of the bonding temperature on the microstructure of the joint was analyzed and the results showed that the nano Ti/Al multilayer foils are promising interlayers for diffusion bonding of TiAl at lower temperatures. The TEM observations for the sample bonded at 1000 °C are given in Figure 8 [82], which revealed a sound joint without unbounded regions at the multilayer/substrate and multilayer/multilayer interfaces. The nanocrystalline grains with the sizes ranging from 50 to 300 nm were observed and no Ti and Al layered structures remained indicating sufficient reaction between Ti and Al. EDX analyses show that the interface region mainly consisted of γ-TiAl grains and a few α_2_-Ti_3_Al grains, which was in accordance with the selected area diffraction pattern of the interface grains. The additional experiments on diffusion bonding of TiAl using Ni/Al multilayer were successfully carried out and the results proved that the Ni/Al multilayer was also suitable for diffusion bonding of TiAl [73,76,77]. Because the bonding quality was closely related to the reaction heat generated by the Ni/Al multilayers, the highest strength values reduced abruptly when the period increased from 14–30 nm.

### 3.2. Diffusion Bonding of Titanium Aluminides to Other Materials

The realization of joining titanium aluminides to other materials opens up the possibility of producing dual property components like compressor and low pressure turbine blisks [85]. However, the diffusion bonding of titanium aluminides to other materials is more difficult than bonding to themselves. When titanium aluminides were diffusion bonded to other materials, it was necessary to optimize the interfacial reaction products and reduce the thermal stresses, especially for joining with ceramics, as demonstrated in [66,68,86].

**Figure 9:**
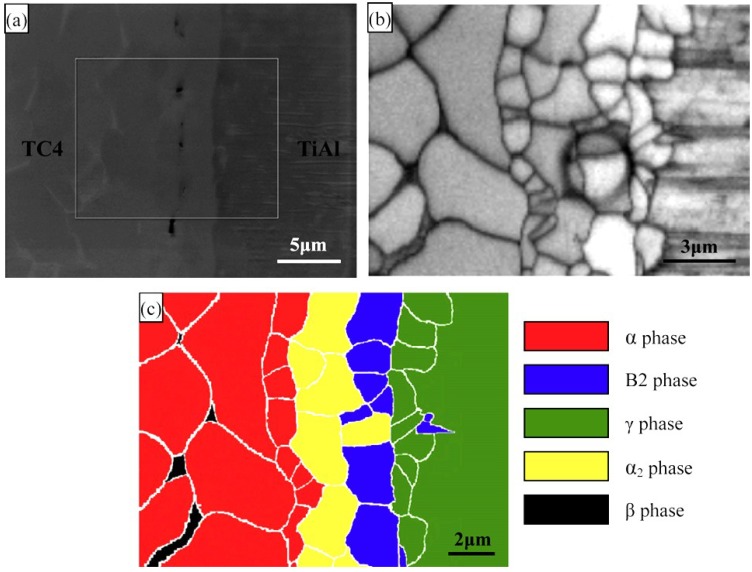
(**a**) Electron backscatter diffraction (EBSD) analysis field of the as-prepared joint interface; (**b**) the front backscatter electron micrograph of the EBSD analysis field in (**a**); (**c**) the schema of phase distribution in the EBSD analysis field. (Reprinted with permission from [69]. Copyright 2013 Elsevier.)

The diffusion bonding between titanium aluminides and Ti alloys was systematically investigated in the existing literature [67,69,85,87,88]. According to elemental gradient diffusion at the interface, the preferred reaction phase betwen TiAl intermetallics to TC4 alloys is α_2_-Ti_3_Al phase. Wang *et al.* [69] reported that the interfacial phase sequence of the diffusion bonded joint was identified as Ti-43Al-9V/γ-TiAl/B_2_/α_2_-Ti_3_Al/α-Ti/Ti-6Al-4V, as shown in Figure 9 [69]. Kanai *et al.* [87] also confirmed the formation of Ti_3_Al phase when diffusion bonding TiAl to Ti-17 alloys. Holmquist *et al.* [85] analyzed the tensile and creep properties of diffusion bonded IMI 384 titanium alloy to IHI 01A γ-TiAl joint. The yield stresses of the joint bonded at 900 °C/200 MPa/3.6 ks are slightly higher in comparison with the γ-TiAl substrate at room temperature, which indicated that sound joints with good mechanical properties can be achieved by diffusion bonding. Although the microstructural observation revealed that larger discontinuity in chemical composition and mechanical properies existed between TiAl substrate and the bond line, the strength of the bond line was high enough to make the fracture initiation process a competition between interfacial initiation and initiation in the TiAl substrate. A large lamellar colony with an unfavourable orientation or a large γ-grain at the surface can act as a defect and promote earlier crack initiation, which leads to the variation in fracture location.

When the titanium aluminides were diffusion bonded to steels, careful control of the bonding parameters was needed to avoid the deterious growth of the brittle compounds at the interface. Mirizono *et al.* [89] realized the diffusion bonding of TiAl intermetallics to eutectoid steels. The reported interfacial microstructure consisted of four kinds of reaction layers: two layers with Ti, Al and Fe elements, TiC layers containing Fe-Al compounds and ferrite layer. The TiAl/steel joint bonded at 1073 K for 3.6 ks has the highest bonding strength of 160 MPa. However, the increase of the bonding tempature led to a dramatic decrease of strength, which is attributed to the growth of the reaction layer and the formation of microcracks at the interface. In order to optimize the interfacial reaction products, a Ti/V/Cu composite barrier layer was added in the TiAl/steel diffusion bonded joint [90]. At the TiAl/Ti interface, a dual phase Ti_3_Al + TiAl layer and a Ti solid solution was formed, which was similar with the TiAl/TC4 bonded joint. No obvious reaction layers were found at the V/Cu and Cu/steel interface. The brittle Ti-Al-Fe and TiC compounds were prevented and the strength of the joint (1273 K/1.2 ks/20 MPa) reached 420 MPa. Gong *et al.* [91] carried out the first principles calculations of Pd/TiAl interface and revealed that the coherent Pd/TiAl interfaces had high values of bond strength. The results confirmed that the TiAl intermetallics was a promising support for Pd membranes.

As one of the candidates to replace the superalloys, the coating technique of titanium aluminides is important for application as turbine blades and vanes. SiC is considered to be adequate for coating TiAl, which requires clarifying the bonding mechanism between TiAl alloys and SiC ceramics. Tenyama *et al.* [68] has realized the diffusion bonding of SiC to TiAl and the interfacial phase sequences of the joints were SiC/TiC/(TiC + Ti_2_AlC)/(Ti_5_Si_3_C*_x_* + Ti_2_AlC)/TiAl_2_/TiAl at room temperature. Liu *et al.* [86] also reported the similar interface structure of SiC/TiC/(Ti_5_Si_3_C*_x_* + TiC)/TiAl and the thickness of each reaction layer increased with the bonding time according to a parabolic law. The deterious growth of continuous reaction layer tended to produce large thermal stresses and impair the mechanical properties of the joint. In order to prevent the formation of the brittle compounds, the Ti/Ni [92] and Zr/Ni [93] barrier interlayers were added during diffusion bonding of TiAl alloys to Ti_3_AlC_2_ ceramics. Optimizing the bonding parameters, the shear strength of the joint bonded at 920 °C/30 MPa/3.6 ks reached 151.6 MPa. The fracture took place in the reaction intermetallic layer adjacent to Ti_3_AlC_2_ substrate, which was consistent with the results of microhardness distribution across the joint.

**Figure 10:**
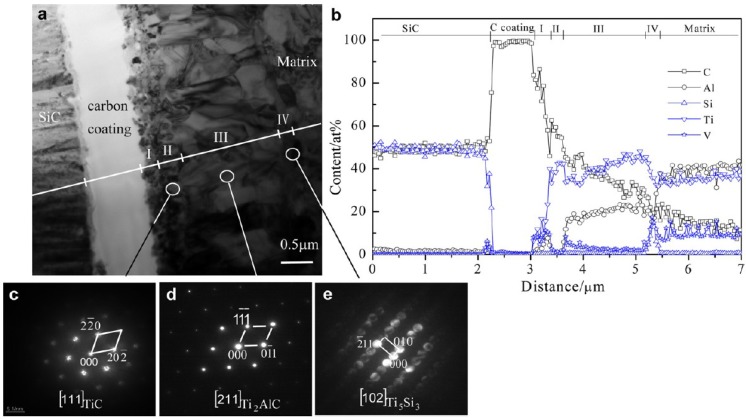
(**a**) TEM morphology of interfacial reaction zone between SiC fiber and TiAl matrix; (**b**) Composition distribution profile across the interfacial reaction zone and electron diffraction patterns of (**c**) TiC in layer II; (**d**) Ti_2_AlC in layer III and (**e**) Ti_5_Si_3_ in layer IV. (Reprinted with permission from [96]. Copyright 2013, Elsevier.)

The reaction between TiAl and ceramic phase has obtained great interest with the development of TiAl matrix composites [94,95,96]. Paransky *et al.* [97] reported the reactive phase formation at the AlN/TiAl interlayer at temperatures ranging from 900–1100 °C. At 900 and 1000 °C no reaction was observed. When holding at 1100 °C, the ternary Ti_2_AlN and Al-rich TiAl phases were formed at the interface between AlN and TiAl. The growth of Ti_2_AlN layer, which obeys the parabolic law, is very low and the thickness was less than 3 μm after a 108 ks annealed at 1100 °C. The interfacial reaction between SiC and TiAl was analyzed by TEM (as shown in Figure 10) and the results indicated that three types of reaction products TiC, Ti_2_AlC and (Ti,V)_5_(Si,Al)_3_ are generated at the interface. The TiC layer can be divided into fine-grained layer and coarse-grained layer [96]. Djanarthany *et al.* [94] also confirmed the following phases of TiC, Ti_2_AlC and Ti_5_Si_3_C*_x_* at Ti-48Al-2V/SCS-6 interface. For the reaction at Ti_2_AlNb/Al_2_O_3_ interface, the decomposition of Al_2_O_3_ occurred and the oxygen diffusion into the substrate to form TiO when vacuum annealing at 750 °C [98].

In general, the mechanical properties of titanium aluminides to other materials diffusion bonded joint are listed in Table 5. The results indicated that the joint between TiAl and other metal tended to obtain relatively higher strength compared with the joint between TiAl and ceramic. Especially when TiAl was bonded to titanium alloys, the joint strength was comparable to the TiAl substrate [85,87,99]. The main reason is that the difference in physical and chemical properties between TiAl and metal is lower. In addition, the interfacial reaction products similar with the TiAl substrate usually formed. However, for TiAl/ceramic diffusion bonded joint, the continuous and thick brittle reaction layers tended to be generated, for example (Ti_5_Si_3_C*_x_* + TiC) layer in TiAl/SiC joint [86,100], which led to the residual stress concentration and degradation of the joint properties.

**Table 5:** Mechanical properties of titanium aluminides/other materials diffusion bonded joint.

Substrate	Interlayer	Joint Strength (MPa)	Bonding Parameters	Fracture Position	References
IMI 384 Ti alloy/IHI 01A γ-TiAl	–	>400 *	900 °C/200 MPa/3.6 ks	Interface	[85]
TiAl/Ti-17	–	>410 *	1000 °C/9.8 MPa/3.6 ks	TiAl substrate	[87]
Ti-36Al/Eutectoid Steel	–	160	800 °C/3.6 ks	Interface	[89]
TiAl/Steel	–	420 *	1000 °C/20 MPa/1.2 ks	TiAl_3_ and TiAl layer	[90]
Ti-46Al-2Cr-2Nb/Ti3AlC2	Ti/Ni foils	151.6	920 °C/30 MPa/3.6 ks	Intermetallic layer	[92]
	Zr/Ni foils	103.6	850 °C/30 MPa/3.6 ks	Ceramic substrate	[93]
Ti-46.5Al-2.5V-2Cr-1.5Nb/Ti-6Al-4V	–	BSR > 80% #	900 °C/100 MPa/7.2 ks	Outside of bonding zone	[99]
Ti-43Al-1.7Cr-1.7Nb/SiC	–	240	1300 °C/35 MPa/900 s	(Ti_5_Si_3_C*_x_* + TiC) layer	[100]
Ti-37.8Al-1.4Cr-1.4V/GH99	Ti6Al4V-0.5H foil	259	850 °C/20 MPa/1.8 ks	Bonding zone	[101]

* tensile strength; # Three-point bending strength, the other values are shear strength. BSR-the ratio of bending strength of the joints to that of TiAl substrate.

## 4. Fusion Welding of Titanium Aluminides

The industrial application of titanium aluminides will inevitably be limited if they can not be welded effectively. Although it is difficult to control the parameters, the fusion welding of titanium aluminides has attracted more and more interest considering the structural size and application temperature of the weld [19,20,102,103,104,105]. However, titanium aluminides, especially for gama-TiAl based intermetallics, have been reported as having limited weldability because of the intrinsic brittleness and poor damage tolerance [106].

According to the published literatures, electron beam welding (EBW) is the most popular method for fusion welding of titanium aluminides and quite a few successful experiments have been carried out [16,20,102,103,104,107,108,109]. Because of the difference in the mechanical properties of the substrates, the electron beam welding of TiAl based intermetallics is much more difficult than that of Ti_3_Al or Ti2AlNb based intermetallics. For example, the room temperature tensile strength of the Ti-22Al-25Nb/TC11 EBW joint is higher than that of the TC11 substrate [108]. However, the tensile strength of the TiAl/TC4 EBW joint is only 411.3 MPa, which is much lower than that of Ti_3_Al/TC4 joint (831 MPa) [104,110].

For electron beam welding of titanium aluminides, the microtructure evolution and optimization of welding parameters was systematically investigated. The solid-state cracking is the main problem for electron beam welding of TiAl. The prior literatures [20,102,103,107,109] demonstrated that the susceptibility of the titanium aluminides to solid-state cracking was attributed to the phase structure and the concentration of residual thermal stressess.

For the small-size workpieces, the effect of the phase transformation on the cold cracking is dominant. The preferred welding parameters should ensure that the α → γ phase transformation will not be suppressed and the welds consisting of γ phase and γ/α_2_ lamellar phase structure are obtained. If the welds mainly contained a fully Al-supersaturated α_2_ phase, which is extrmely brittle and undesirable, the solid-state cracking tends to be generated during the EBW process [103,109].

**Figure 11:**
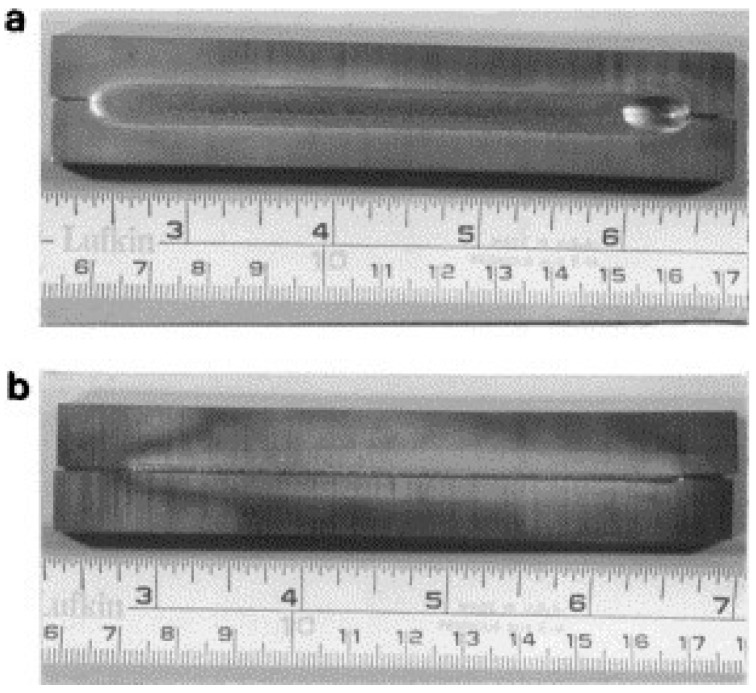
(**a**) Top and (**b**) bottom views of a crack-free weld made at a cooling rate of 240 °C·s^−1^. (Reprinted with permission from [107]. Copyright 2001 Elsevier.)

For highly restrained workpieces, the role of phase decomposition was overshadowed by the residual thermal stresses and the TiAl alloys becomes extremely difficult to weld [107]. Therefore, very careful control of welding parameters is required for electron beam welding of TiAl alloys. Chaturvedi *et al.* [102,107] analyzed the effect of the cooling rate and concluded that the critical cooling rate at the fusion zone boundary is 250 °C·s^−1^ for the Ti-45Al-2Nb-2Mn + 0.8 vol% TiB_2_ alloy. To test the results, the EBW experiment made at a cooling rate of 240 °C·s^−1^ was carried out and a crack-free butt was obtained, in which the top and bottom views of weld are shown in Figure 11 [107].

Based on a similar consideration, a novel composite control method was developed to obtain a reliable EBW joint with a tensile strength of 411.3 MPa [103], which was related to the prolonging the high temperature stage by heat accumulation and compensation. Additionally, the alloying element also has a strong effect on the electron beam welding of titanium aluminides. The addition of niobium element (5–8 wt%) has a positive effect on the strength, creep and oxidation resistance [20]. The Ti-48Al-2Nb-2Mn alloy was found to be less susceptible to cold cracking than that of Ti-45Al-2Nb-2Mn alloy because the α phase in the alloy with a higher Al content could decompose more readily [109].

**Figure 12:**
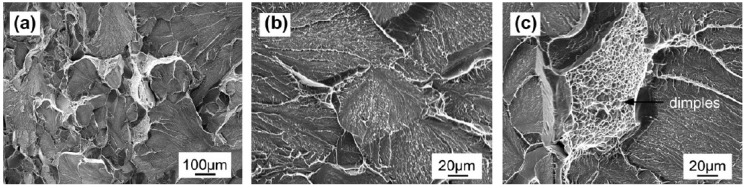
SEM fractograph of the joint after tensile test at room temperature: (**a**) over view; (**b**) cleavage planes and (**c**) ductile dimples in the cleavage steps. (Reprinted with permission from [105]. Copyright 2013 Elsevier.)

As a high power-density method, laser welding of titanium aluminides was also performed and the crack-free joint was obtained [19,105,106]. Similar with electron beam welding of TiAl intermetallics, the conventional laser welding process consists of three steps: pre-heating, welding and post-weld heat treatment, which aims at decreasing the cooling rate. If the pre-heating temperature was higher, the higher welding speed was feasible. The pre-heating process can be performed by induction heat [106] or cartridge heat [19]. Kuwahara *et al.* [19] examined the relationship between pre-heating temperature and welding speed. The welding speeds with full bead-on-plate welding were strongly dependent on the initial pre-heating temperature: 50 mm/s at initial temperature 600 °C, 43 mm/s at initial temperature 400 °C and 33 mm/s at initial temperature 27 °C [19]. In addition, the protection of the Ti-Al substrate from oxidation is necessary for laser welding [19,105,106]. Liu *et al.* [106] established an experimental step-up including an induction furnace and an oxygen measuring facility. The laser welding was carried out in the furnace with argon to protect the TiAl plates from oxidation. At the same time, flow helium was used as the assistant gas supplied by an off-axis nozzle to the weld zone. The residual oxygen content in the chamber was continuously measured, and the welding process was performed after the oxygen content was below 100 ppm. The tensile strength of the Ti-22Al-27Nb joint was comparable to that of the base metal and the tensile ductility of the joints reached 56% of the base metal. The corresponding photograph exhibits quasi-cleavage fracture characteristic, as shown in Figure 12 [105]. The fracture surfaces represented the cleavage planes and tearing ridges or dimples.

As mentioned above, the high power-density welding methods are suitable for welding of titanium aluminides. However, the traditional arc welding is very difficult for obtaining a crack-free joint. The effect of rolling direction on the microhardness and microstructure of the TiAl joint by gas tungsten arc welding was examined by Acoff *et al.* [18]. The immediately post-weld heat treatment of 615 °C for 7.2 ks was necessary to relieve the internal stresses. The results showed that the specimens welded transversely to the rolling direction are more sensitive to cold crack than that in the direction of rolling. Although the strict welding parameters are required, the prospect of fusion welding of titanium aluminides is promising due to the highly convenient and efficient welding process.

## 5. Joining of Titanium Aluminides by Other Methods

Taking the successful diffusion bonding results into account, the friction welding of titanium aluminides was performed as a more efficient solid-state bonding method [12,26,111,112]. The effect of welding parameters on microstructure and mechanical properties are examined and analyzed. It is noted that the cracks and voids are the main defects in the TiAl joint despite of the unmelted substrate. Because of the TiAl side local grain refinement and the locally different microstructual orientaions, the isotropy degree increased in a local region when the friction welding of TiAl to Ti6Al4V was carried out [112]. In particular, the local mirohardness was primarily determined by the grain size and not by the phase distribution.

**Figure 13:**
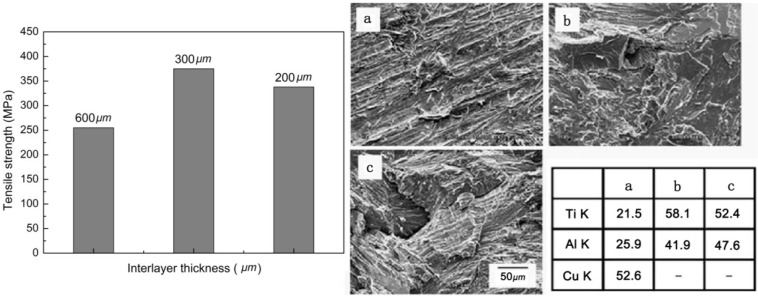
Tensile strength variations with various insert layer thickness, SEM fracture surface (**a**) 600 mm; (**b**) 300 mm; (**c**) 200 mm and EDS analysis. (Reprinted with permission from [26]. Copyright 2004, Elsevier.)

Although TiAl and steel has been successfully joined by diffusion bonding and brazing, the requirements for vacuum equipment, shield gas and filler metal limit the application in the industry fields. Based on this, the friction welding of TiAl to steel presented many process advantages due to the specific joint characteristics for joining dissimiliar materials [12,26]. When the TiAl was directly friction welded to AISI 4140, the crack was generated at the interface and the tensile strength was only 120 MPa because of the brittleness of the reaction products. When a pure copper interlayer was inserted between the TiAl and AISI 4140, the crack formation and martensite transformation was prevented and the highest tensile strength of 375 MPa was obtained using the interlayer with a thickness of 300 μm (Figure 13) [26]. While the fracture took place at the interface when the 600 μm interlayer was used, the fracture occurred in the TiAl substrate in the case of the 200 and 300 μm interlayers.

Resistance spot welding is the most widely used method in the joining of thin metal sheets due to its high effeciency and high degree of automation, in which a small weld is formed between two metal workpieces through localized melting due to interfacial resistance heating. The present study revealed that the spot resistance welding of titanium alloys to themselves and to other materials was successfully performed [113,114,115,116,117,118,119,120]. Especially, the interfacial microstructure mainly consisted of TiAl_3_ intermetallics when the the titanium alloys were spot resistance welded to aluminium alloys [116,120], which indicated the possibility of spot resistance welding of titanium aluminides. The dissimilar alloy welds have been produced between Ti-6Al-2Sn-4Zr-2Mo-0.1Si (wt%) and Ti-13.5Al-21.5Nb (wt%) titanium aluminide using capacitor discharge resistantce spot welding [119]. The extremely rapid heating of the weld interface region to near-solidus temperature and subsequent rapid cooling led to the formation of a metastable ordered-beta microstructure in the Ti-13.5Al-21.5Nb and fine alpha-prime martensite in Ti-6Al-2Sn-4Zr-2Mo-0.1Si substrate. The resistance spot welding of Ti_2_AlNb was carried out and serious reheat cracking susceptibility was found [113]. The reheat cracking occurred from about 500 °C. When the temperature was sufficiently low (450 °C), the specimen did not crack even after holding for 7.2 ks. On the other hand, when the temperature was increased to 550 °C, the cracking occurred in less than 600 s. Therefore, it was concluded that the cracking was more susceptible to temperature than hold time. With the increase of temperature, the precipitation of O phase impaired the hot ductility and the residual stresses were concentrated, which led to the increase of the crack susceptibility.

Recently, several attempts have been made to reactive joining of titanium aluminides, which involves placing a reactive interlayer between the two bonded surfaces and igniting the exothermal reaction as a local heat source [27,28,121,122,123,124]. The joining heat was generated by the reaction in the powder mixtures or multilayer interlayers. The design of the reaction interlayer, which determined the heat generated by the combustion synthesis, is the fundamental step for reactive joining. If the reaction heat is insufficient, the interfacial reaction and joining can not be realized. However, if the reaction heat was too high, the deleterious reaction phases tend to form and the residual thermal stresses are large, which also leads to the impairment of the joint strength. Therefore, the adiabatic temperature is calculated to evaluate the reaction heat in the interlayer. Uenishi *et al.* [28,61] successfully carried out the self-propagating high-temperature synthesis joining of TiAl using a Ti + Al powder mixture as an interlayer. The Ti-Al powder mixture was compacted to a relative density of 85% and the compact was inserted between two TiAl based metals. The interfacial microstructure of the joint contained a TiAl_3_ layer with a thickness of 10 μm. The structure of the reaction products in the interlayer was not lamellar and comprised TiAl_3_ and unreacted Ti. Subsequent heat treatment at 1300 °C for 10.8 ks changed the interlayer structure to γ/α_2_ lamellar with a lamellar size of ~50 μm, which was much smaller than that of the based metal. The tensile strength of the TiAl joint was 220 MPa, which was almost the same as that of the based metal at room temperature and 600 °C.

**Figure 14:**
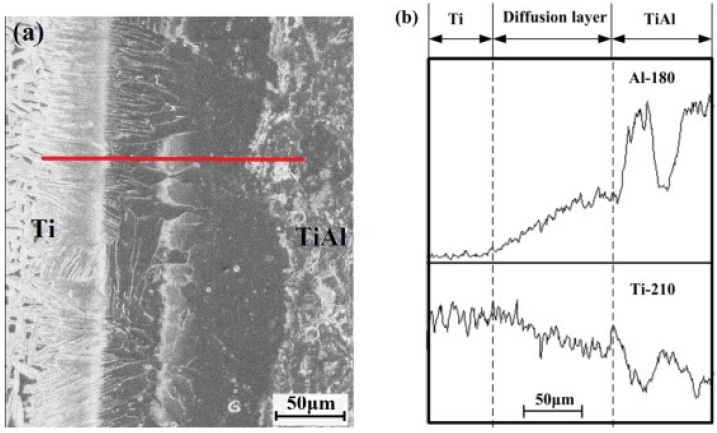
(**a**) Microstructure of the interface between TiAl/Ti and (**b**) EDS analysis across the interface. (Reprinted with permission from [125]. Copyright 2012, Elsevier.)

Because the TiAl intermetallics were successfully manufactured by combustion synthesis, the material preparation and joining operation could be simplified into a single step if the C/C composites could be simultaneously joined to the as-synthesized TiAl during the combustion synthesis process [122]. For reactive joining of TiAl to other materials, the investigations paid attention to the control of interfacial reaction and the release of residual stresses in the joint. The microstructural results showed that the interfacial compressive stresses were beneficial for ascertaining the most reliable joining [122]. Chen *et al.* [124,125] also succesfully fabricated TiAl by combustion systhesis and simultaneously joined the as-synthesized TiAl to Ti and TiC-Ni substrate. The 64% Ti and 36% Al (wt%) powders were mixed and cold pressed in a graphite die with a diameter of 20 mm. A direct current of 1400 A was applied for 900 s to stimulate the reaction and bonding. The microstructure shown in Figure 14 at the Ti/TiAl interface was : pure Ti, Ti(Al) solid solution, Ti_3_Al, Ti_3_Al + TiAl mixture, and TiAl.

Another important application for joining of TiAl by combustion synthesis is to fabricate a protective coating on Ti alloy and steel substrates [126,127,128,129]. The mixed Ti-Al coating powders were put on the suface of the substate. Then the specimen was heat treated at high temperature to ensure the sufficient combustion reaction in Ti-Al system to form TiAl and simultaneously join with the substrate. Romankov *et al.* [126,127] deposited Ti + Al coatings (with a thickness of 200 μm) on Ti alloy substrates by means of the mechanical alloying method. Annealing treatment at temperature ranging between 600 and 1100 °C ignited the reaction between Ti and Al to form different Ti-Al intermetallics on the samples. In the case of Ti + Al coating, Al_3_Ti, Al_2_Ti, TiAl and Ti_3_Al were formed on the surface of Ti substrate. The coating annealed at 1100 °C was very dense, and no peeling, microcracks or pores were observed, which indicated the sound joining between Ti-Al intermetallic coatings and Ti substrates.

## 6. Conclusions

In this review article, current developments in microstructure and properties of titanium aluminide joints, which were obtained by brazing, diffusion bonding, fusion welding, friction welding and reactive joining, have been addressed.

Brazing is an effective method for joining titanium aluminides using Ti-based, Ag-based and Al-based filler metal. The Ti-Cu-Ni filler metal was the most commonly used Ti-based filler metal for infrared brazing and furnace brazing. When TiAl was brazed using this filler metal, the α-Ti and α_2_-Ti_3_Al phase tended to form at the interface, and the brazed seam mainly consisted of Ti-rich and Ti_2_Ni phase. The highest strength of joint using Ti-based filler metal reached 373 MPa and the fracture mainly propagated along the Ti_2_Ni layer. The Ag-Cu-Ti filler metal was the most commonly used Ag-based filler metal, in which the interfacial reaction phases were mainly composed of AlCuTi and AlCu_2_Ti phases. The joint properties using Ag-based filler metal were satisfied, which derived from the ductile Ag-rich solid solution in the brazed seam to buffer the residual stress. When BAlSi-4 filler metal was used, the highest shear strength was 86.2 MPa and the formation of brittle Ti-Al-Si ternary compounds resulted in the low strength of TiAl brazed joint. The control of the brittle interfacial compounds was the key factor related to the joint properties.

Diffusion bonding was also a widely used method for joining titanium aluminides. When titanium aluminides were bonded to themselves, the main defects were unbonded regions at the interface. The post bond heat treatment is preferred and the strength of the joint was comparable to the substrate. The application of nano Ni/Al multilayer as interlayer was beneficial for decreasing the bonding temperature and improving the bonding quality. The diffusion bonding of titanium aluminides to other materials is more difficult than bonding to themselves. When TiAl was bonded to Ti alloys, the Ti_3_Al phase was dominant at the interface. When TiAl was bonded to steel, the addition of a Ti/V/Cu composite barrier layer was effective to prevente the brittle Ti-Al-Fe and TiC compounds and improve the strength of the joint to 420 MPa. The Ti_5_Si_3_C*_x_* + Ti_2_AlC phases were generated at the interface of the TiAl/SiC joint, which led to the residual stress concentration and degraded the joint properties.

Electron beam welding and laser beam welding were the two effective methods for fusion welding of titanium aluminides. The solid state crack was the main defect for fusion welding of titanium aluminides, which meant that careful control of welding parameters was required. The preheating and post weld heat treatment was usually necessary to reduce the cooling rate and obtain the expected microstructure. The preferred welding parameters should ensure the sufficient α → γ phase transformation and obtain the welds consisting of γ phase and γ/α_2_ lamellar phase structure. The conventional laser welding process consisted of three steps: pre-heating, welding and post-weld heat treatment, which also aimed at decreasing the cooling rate. The surface protection was necessary for the laser beam welding of titanium aluminides. The tensile strength of the Ti-22Al-27Nb joint was comparable to that of the base metal and the tensile ductility of the joints reached 56% of the base metal.

The titanium aluminide joint using friction welding tended to fracture due to the cracks and voids. The addition of Cu interlayer was an effective way to optimize the interfacial reaction products and reduce the residual stresses, which finally improved the joint strength to 375 MPa using the Cu interlayer with a thickness of 300 μm. The reactive joining of titanium aluminides was also successfully performed and the microstructure characteristic was introduced. The interfacial microstructure of the joint contained a TiAl_3_ layer with a thickness of 10 μm and the tensile strength of the TiAl joint was almost the same as that of the based metal at room temperature and 600 °C.
